# Transcriptome analysis reveals the molecular mechanism of differences in growth between photoautotrophy and heterotrophy in *Chlamydomonas reinhardtii*

**DOI:** 10.3389/fpls.2024.1407915

**Published:** 2024-06-19

**Authors:** Jing Chen, Yuanhao Chen, Weiling He, Honghao Liang, Ting Hong, Tangcheng Li, Hong Du

**Affiliations:** ^1^ Guangdong Provincial Key Laboratory of Marine Biotechnology, STU-UNIVPM Joint Algal Research Center, Institute of Marine Sciences, Shantou University, Shantou, Guangdong, China; ^2^ Southern Marine Science and Engineering Guangdong Laboratory, Guangzhou, Guangdong, China; ^3^ Guangdong Provincial Key Laboratory of Marine Disaster Prediction and Prevention, Shantou University, Shantou, Guangdong, China

**Keywords:** *C. reinhardtii*, photoautotrophy, heterotrophy, transcriptome, metabolic pathways

## Abstract

**Background:**

The green alga *Chlamydomonas reinhardtii* can grow photoautotrophically utilizing light and CO_2_, and heterotrophically utilizing acetate. The physiological and biochemical responses of autotrophy and heterotrophy are different in *C. reinhardtii*. However, there is no complete understanding of the molecular physiology between autotrophy and heterotrophy. Therefore, we performed biochemical, molecular and transcriptome analysis of *C. reinhardtii* between autotrophy and heterotrophy.

**Results:**

The cell growth characterization demonstrated that heterotrophic cell had enhanced growth rates, and autotrophic cell accumulated more chlorophyll. The transcriptome data showed that a total of 2,970 differentially expressed genes (DEGs) were identified from photoautotrophy 12h (P12h) to heterotrophy 12h (H12h). The DEGs were involved in photosynthesis, the tricarboxylic acid cycle (TCA), pyruvate and oxidative phosphorylation metabolisms. Moreover, the results of qRT-PCR revealed that the relative expression levels of malate dehydrogenase (MDH), succinate dehydrogenase (SDH), ATP synthase (ATPase), and starch synthase (SSS) were increased significantly from P12h and H12h. The protein activity of NAD-malate dehydrogenase (NAD-MDH) and succinate dehydrogenase (SDH) were significantly higher in the H12h group.

**Conclusion:**

The above results indicated that the high growth rate observed in heterotrophic cell may be the effects of environmental or genetic regulation of photosynthesis. Therefore, the identification of novel candidate genes in heterotrophy will contribute to the development of microalga strains with higher growth capacity and better performance for biomass production.

## Introduction

As we all know, microalgae are found in oceans and lakes around the world. They are abundant in vitamins, fatty acids, proteins, and several nutrients, which are important food sources for fish, shellfish, and other animals (Spolaore et al., 2006; Wulff et al., 2009). Microalgae are typically autotrophic organisms that depend on organic and inorganic carbon sources for cell growth (Li et al., 2020). Phototrophic microalgal species can produce energy and carbon for growth using inorganic compounds and light. Heterotrophic microalgal species are wholly dependent on organic material for nutrition (Caron, 2016). In addition, a small proportion of microalgae can be both photoautotrophic and heterotrophic (Markou, 2020). Some microalgal species cannot utilize organic carbon to grow. A deficiency of glucose-concentrating capacity was demonstrated in three cases: *Nostoc* sp. (PCC7118) and two *Synechocystis* strains (PCC6308 and PCC7008) (Zhang et al., 1998). *Spirulina* sp. was able to grow photoautotrophically, heterotrophically, and mixotrophically (Chojnacka and Noworyta, 2004). *Chlorella vulgaris* was able to grow in both autotrophic and heterotrophic conditions. While autotrophic growth provided higher cellular lipid content, the lipid productivity was much lower compared with heterotrophic growth (Liang et al., 2009). The green alga *C. reinhardtii* can grow photoautotrophically utilizing CO_2_, heterotrophically utilizing acetate, and mixotrophically utilizing both carbon sources (Heifetz et al., 2000). *C. reinhardtii* is an excellent model organism in study photosynthesis, cell physiology, and photoprotective mechanisms (Wakao et al., 2021). *C. reinhardtii* was photoautotrophic through light-related metabolic pathways, such as photosynthesis, porphyrin, chlorophyll metabolism, and carotenoid biosynthesis. When *C. reinhardtii* was cultured under heterotrophic conditions, they mainly showed dark hypoxic metabolism and acetic acid metabolism (Boyle and Morgan, 2009a).

With the rapid development of omics, physiological data and omics-based technologies, such as transcriptomics, metabolomics, and proteomics have been increasingly used cooperatively to provide mechanistic insights into growth differences of microalga in different cultivation conditions (Vidotti et al., 2020). Transcriptome sequencing (RNA-seq) is a cost-effective and time-saving approach to survey the putative differentially expressed genes (DEGs) and contribute to understanding biological processes and metabolic pathways of microalgae from autotrophic to heterotrophic conditions (Vidotti et al., 2020; Ma et al., 2023). Transcriptome analysis contributed to finding target genes and pathways that could be the focus of further physiological and molecular studies (Dong et al., 2023). Recent studies have summarized advances in transcriptomic research on heterotrophic and autotrophic protists, including physiology and metabolism, development and life cycles, environmental and ecological studies (Anderson, 2022a).

Previous studies demonstrated that *C. reinhardtii* mainly fed on cells during photoautotrophy through light-related metabolic pathways, such as photosynthesis, porphyrin, chlorophyll metabolisms, and carotenoid biosynthesis. When *C. reinhardtii* was cultured under heterotrophic conditions, they mainly showed dark hypoxic metabolism and acetic acid metabolism (Heifetz et al., 2000). However, little information is available on dark oxygen metabolism in *C. reinhardtii*. Dark culture conditions can accumulate more triacylglycerol (a type of fat) in *C. reinhardtii*, which have potential applications for biofuel production (Liang, 2013). Therefore, it is critical to investigate mechanisms by which *C. reinhardtii* can adapt to these transitions, with focus on how the cell alter pathways, such as glycolysis (gluconeogenesis), the TCA cycle, lipid metabolism, energy storage, and cell growth (Liang, 2013; Hu et al., 2018). Additionally, during changes in cellular physiology of *C. reinhardtii*, several enzymes are notable for playing key roles in the complex network of dark metabolism between photoautotrophic and heterotrophic cultures (Eriksen, 2008; Morales-Sánchez et al., 2015). However, there is limited research that focus on the transcriptome to compare growth differences of *C. reinhardtii* under autotrophic and heterotrophic conditions. This study attempted to identify molecular alterations by combining physiological, transcriptomic, and molecular methods from autotrophy to heterotrophy. The ultimate goal was to elucidate the molecular basis for growth differences between photoautotrophy and heterotrophy in *C. reinhardtii* and to provide a theoretical basis for the in-depth research on photoautotrophy and heterotrophy in alga. Additionally, candidate genes for transcriptome screening in heterotrophy will contribute to development of microalga strains with higher growth capacity and better performance for biomass production.

## Materials and methods

### Cell strain and culture conditions

*C. reinhardtii* strain CC-125 was obtained from the Chlamydomonas Resource Center (CRC, University of Minnesota). For photoautotrophy, CC-125 cell was continuously cultured in a high-salt (HS) medium (Sueoka, 1960), and heterotrophy was cultured in tris–acetate–phosphate (TAP) medium (Gorman and Levine, 1965) in 500 mL Erlenmeyer flasks (with 150 mL medium). The air was bubbled into the medium at 0.4 L/min using an LZB-3WB float-type flowmeter (Nanjing Shunlaida Measurement and Control Equipment Co., Ltd), which was required for photoautotrophy. Photoautotrophy was cultured under a temperature of 25 °C with a light intensity of 80 μmol m^-2^ s^-1^, a 24:0 h (light:dark) photoperiod, and constant shaking at 120 rpm. By contrast, the culture condition of heterotrophy was the same as above except for the light time, which was a 0:24 h (light:dark) photoperiod.

### Growth measurement and morphological observation

The cell density of *C. reinhardtii* was measured by cell counting or optical density methods between autotrophic and heterotrophic cultures. Chlorophyll was extracted using an 80% acetone solution and the concentrations of chlorophyll a (Chl a), chlorophyll b (Chl b), and total chlorophyll were measured using spectrophotometry as described by Lichtenthaler et al (Harmut, 1987). The following equations (Lichtenthaler and Wellburn, 1983) (Unit: μg/mL) were used: Equations 1–3 were used to calculate chlorophyll a chlorophyll b and total chlorophyll content.


(1) $$\text{Ch a}=12.21\times {\text{A}}_{663}–2.81\times {\text{A}}_{646}$$

(2) $$\text{Ch b}=20.13\times {\text{A}}_{646}–5.03\times {\text{A}}_{663}$$

(3) Ch l=Ch a+Ch b

### RNA quantification and qualification

In the transcriptome analysis, 2×10^7 algal cells cultured in photoautotrophy for 12 hours were collected (for the P12h group). Meanwhile, 2×10^7 algal cells cultured in heterotrophy for 12 hours were collected (for the H12h group). Above cells were transferred into liquid nitrogen for quick freezing and transferred to a -80°C refrigerator for storage. Total RNA was extracted and sequenced by previous methods (Fu et al., 2022). The integrity was assessed using the RNA Nano 6000 Assay Kit of the Bioana-lyzer 2100 system (Agilent Technologies, CA, USA). The transcriptome experiments were performed by Novogene (https://www.novogene.cn/).

### Transcriptome analysis

The clustering of index-coded samples was performed on the cBot Cluster Generation System using the Tru Seq PE Cluster Kit v3-cBot-HS (Illumina). After cluster generation, libraries were prepared for sequencing on the Illumina Nova seq platform, generating 150 bp paired-end reads. Clean data (clean reads) were obtained by removing reads containing read adapters, reads containing ploy-N, and low-quality reads (the number of bases in Ophred<= 20 is more than 50% of the entire read length). FPKM was the expected per kilobase of transcript per million fragments mapped. The statistical methods were provided by the DESeq2 R software (1.20.0). The Benjamini and Hochberg methods for reducing the false discovery rate at 5% were used to modify the obtained P-values. Genes identified by DESeq2 as having differential expression were those with an adjusted P-value of ≤ 0.05.

Cluster Profiler R package was used to implement a gene ontology (GO) enrichment analysis of the differentially expressed genes (DEGs) that were adjusted for genes length bias. We used a corrected P-value of ≤ 0.05 and a |log2 (fold change) | ≥ 1 to evaluate significant genes expression differences. GO keywords were deemed to be significantly enriched by DEGs (Young et al., 2010). The KEGG (Kanehisa et al., 2008) database was used to understand high-level biological system functions and utilities based on molecular data, particularly sizable molecular datasets produced by genome sequencing and other high-throughput experimental technologies (http://www.genome.jp/kegg/).

### Determination of key enzyme activities

The 2×10^7 cells were collected by centrifugation at 3000 g for 10 min and stored at -80 °C. For enzyme activities, we used 5 million cells in 1 mL PBS and were disrupted by sonication. After centrifugation with 10000 g at 4°C for 10 min, the supernatant was removed and kept on ice for testing. Then, we transferred the supernatant into a test tube containing 10 mL of distilled water and shook before use. Key enzyme activities were then detected using the following kits: Rubisco-Bisphosphate Carboxylase/Oxygenase (Rubisco) Activity Assay Kit; NAD-Malate Dehydrogenase (NAD-MDH) Activity Assay Kit; Soluble Starch Synthase (SSS) Activity Assay Kit; Pyruvate Kinase (PK) Activity Assay Kit; Succinate Dehydrogenase (SDH) Activity Assay Kit; ATP Activity Assay Kit.

### RNA extraction and RT-qPCR

The 2×10^7 algal cells were collected and the total RNA was extracted with TRIzol (Takara, Shiga, Japan). The concentration and purity of RNA were assessed using a Nanodrop microspectrophotometer (Thermo Fisher Scientific, Wilmington, DE, USA). After determining the RNA quality using agarose gel electrophoresis, complementary cDNA synthesis was carried out using Rever Tra Ace qPCR RT Master Mix with gDNA Remover (TOYOBO). Quantitative real-time PCR (qRT-PCR) was performed with an Applied Biosystems 7300 Real Time PCR System and a Roche Light Cycler 96 system, using TB Green® Premix Ex Taq™ II to measure the relative transcript levels of genes associated with metabolic changes. The ubiquitin ligase UBC8 (Phytozomev10.2, cre03.g159200.t1.1) was used as the endogenous control (Díaz-Santos et al., 2016). Cycling conditions were 10 min at 95°C with 40 cycles for melting (30 s at 95°C), annealing (30 s at 60°C), and extension (30 s at 72°C). The genes of interest and the primers were listed in **Supplementary Table S6**. The data was quantitatively analyzed by 2^−△△CT^.

### Statistical analysis

The experiments were performed with three or four biological replicates and presented as means ± standard deviation (SD). Statistical analysis was performed using the statistical package “SPSS 20.0 for Windows”. The results of qRT-PCR were calculated as described above. PowerPoint, GraphPad Prism 8, and Paint 3D (Windows 10) were used to draw and modify figures. All data were firstly tested for homoscedasticity and normality. The significance of different treatment groups was analyzed with one-way analysis of variance (ANOVA) and two-way ANOVA, both of which were followed by Tukey’s test, with significance determined at P< 0.05 (*) and P< 0.01(**). Bars are means ± SD (n = 3 or 4).

## Results

### Effects of photoautotrophy and heterotrophy on the growth of *C. reinhardtii*

**Figure 1:**
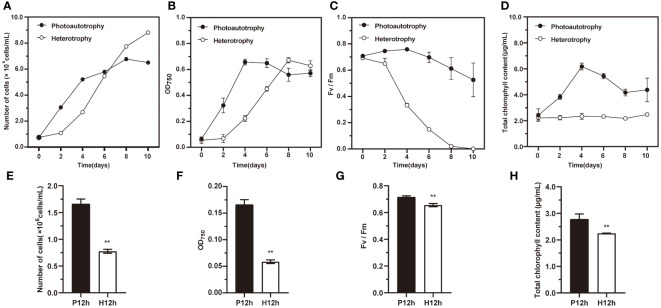
The growth characteristics of *C. reinhardtii* in photoautotrophy and heterotrophy. **(A)** Number of cells cultured under photoautotrophy and heterotrophy at 0, 2, 4, 6, 8, and 10 days; **(B)** OD_750_ variation curves; **(C)** Fv/Fm variation curves; **(D)** Total chlorophyll content; **(E)** Number of cells between P12h and H12h; **(F)** OD_750_ from P12h to H12h; **(G)** Fv/Fm between P12h and H12h; **(H)** Total chlorophyll content between P12h and H12h. Significance was determined by t-test analysis: ** indicated P < 0.01.

As shown in **Figure 1**, the growth characteristics of *C. reinhardtii* in autotrophic and heterotrophic conditions were different, including cell numbers, OD750, Fv/Fm variation curves and total chlorophyll content. The cell numbers increased significantly during ten days in photoautotrophy or heterotrophy (**Figure 1A**). The changes in OD_750_ of *C. reinhardtii* were investigated (**Figure 1B**). It increased significantly to a maximum of 0.66 in photoautotrophy from day 0 to day 4, and then declined slightly over the next few days. By contrast, the heterotrophy increased to its highest point of 0.671 on the eighth day, and then it decreased slowly. The Fv/Fm of photoautotrophy and heterotrophy showed the same downward trend, but heterotrophy was falling faster (**Figure 1C**). The total chlorophyll content of photoautotrophy rose considerably from day 0 to day 4 and then declined gradually in the following days. By contrast, the total chlorophyll content of photoautotrophy did not change abruptly, with variation from 2.4 μg/mL to 2.6 μg/mL (**Figure 1D**). To observe the differences in above indicators between P12h to H12h, we showed bar graphs as in **Figures 1E–H**. The P12h group was significantly higher than the H12h group in the cell numbers, OD750, Fv/Fm, and total chlorophyll content. Photoautotrophy and heterotrophy significantly affected the growth of *C. reinhardtii.*

### Analysis of transcriptome in P12h and H12h

**Figure 2:**
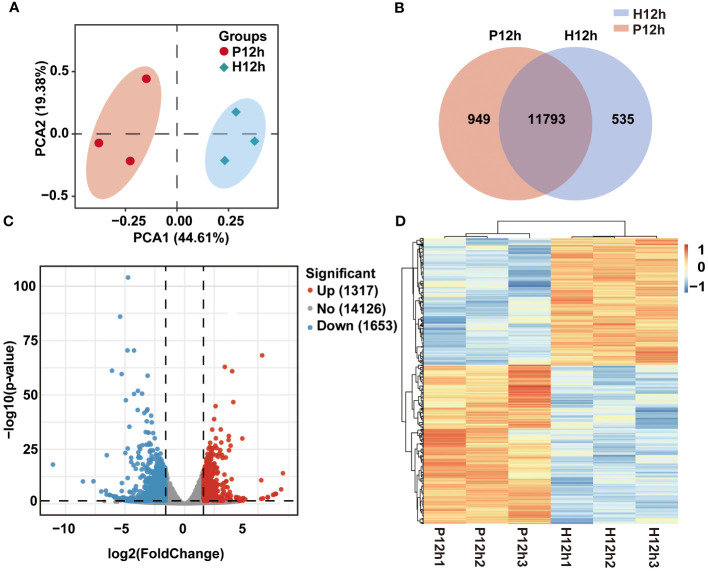
RNA-seq data expression of *C. reinhardtii* in photoautotrophy and heterotrophy. **(A)** PCA analysis, PC1 indicated the first principal component and PC2 indicated the second principal; **(B)** Venn diagram showing expressed genes in P12h vs. H12h; **(C)** The volcanic map of DEGs between P12h and H12h; **(D) **Heatmap analysis of the DEGs in H12h and P12h.

For a better insight into differences of growth, biochemical composition, and photosynthetic physiology of photoautotrophy and heterotrophy in *C. reinhardtii*, transcriptome analysis was used in this study. We constructed total RNA libraries of P12h and H12h by RNA-sequencing (**Figure 2**). According to the Illumina sequencing, the transcriptome sequencing of *C. reinhardtii* produced 39.84–47.81 million raw reads (Illumina NovaSeq 6000). Fastp (version 0.19.7) was used to filter the raw data, obtaining over 37.57 million clean reads (**Supplementary Table S1**). The dependability and credibility of data were shown by a strong correlation coefficient (R^2^ > 0.909) of gene expression between biological replicates (**Supplementary Figure S1**). To assess differences in the sequencing data between P12h and H12h, holistic principal component analysis (PCA) was performed. The first and second principal components explained 44.61% and 19.38% of variation, respectively (**Figure 2A**). The Venn diagram showed the number of uniquely expressed genes in P12h or H12h, and overlapping areas showed the number of co-expressed genes in both groups (**Figure 2B**). The differential genes were screened with p-value ≤ 0.05 and the absolute value of log2 (FC) ≥ 1 as the screening condition. A total of 2970 differential genes (1317 upregulated, 1653 downregulated) were identified between the P12h and the H12h groups (**Figure 2C**). The heat map indicated expression levels of DEGs in the P12h and the H12h groups (**Figure 2D**). DEGs clustered with the R package Mclust demonstrated that the group had similar expression profiles.

### Functional annotations and classification of DEGs

**Figure 3:**
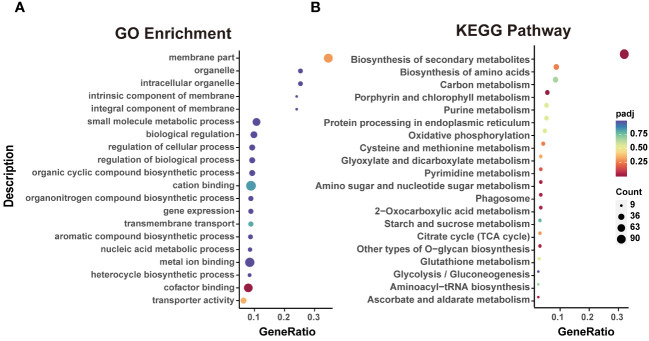
Functional analysis of DEGs. **(A)** Top 20 GO enrichments with DEGs; **(B)** Top 20 KEGG pathways enriched with DEGs.

To explore the regulatory role of DEGs between P12h and H12h, GO annotation and KEGG pathway analyses were performed. In the comparison of DEGs from H12h vs. P12h, the GO analysis showed enrichment of three categories, including Biological Process (BP), Cellular Component (CC), and Molecular Function (MF). They were mainly enriched in ATP hydrolysis and energy transport, including sulfuric ester hydrolase activity, cofactor binding, energy-coupled proton transmembrane transport, and coenzyme binding (**Supplementary Figure S2**). In P12h vs. H12h, the top 20 GO enrichments with DEGs were mainly enriched in membrane, ATP hydrolysis, energy transport cofactor binding, transporter activity and cation binding (**Figure 3A**). Additionally, KEGG pathway enrichment analysis revealed that the majority of DEGs were involved in secondary metabolism (**Supplementary Figure S3**). The top 20 KEGG pathway enrichment analysis demonstrated that DEGs in P12h vs. H12h were mainly enriched in several metabolic pathways, including biosynthesis of secondary metabolites, carbon metabolism, biosynthesis of amino acids, oxidative phosphorylation, citrate cycle, chlorophyll metabolism, and others (**Figure 3B**).

### Analyses of the DEGs related to the photosynthesis and carbon fixation metabolic pathways in P12h and H12h

**Figure 4:**
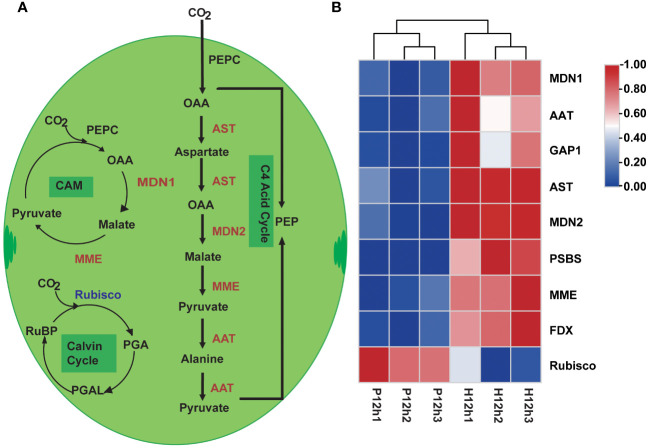
Differentially expressed genes (DEGs) between P12h and H12h were involved in the photosynthesis and carbon fixation metabolic pathways. **(A)** Schematic overview of photosynthesis and carbon fixation in chloroplast of *C. reinhardtii*, PEP: carboxylate phosphoenolpyruvate, PEPC: phosphoenolpyruvate carboxylase, OAA: oxalo acetic acid, Rubisco: ribulose bisphosphate carboxylase, PGA: 3-phosphoglyceric acid, PGAL: 3-phosphoglyceraldehyde, CAM: crassulacean pathway, red words indicated up-regulated DEGs and blue words indicated down-regulated DEGs; **(B)** The heatmap of relative expression of DEGs.

According to the KEGG enrichment analysis, the photosynthesis and carbon fixation metabolic pathways were one of the representative pathways in P12h and H12h. Based on previous studies and transcriptomic data, the simplified photosynthetic carbon fixation metabolic pathway was constructed in **Figure 4A**. The ten DEGs were identified related to the carbon fixation pathway (**Supplementary Table S2**), four of these genes were involved in C4-Dicarboxylic acid cycle, including *AST, MDN2, MME*, and *AAT*. The expression levels of these genes increased from P12h to H12h according to the FPKM values of transcriptome (**Figure 4B**). *MDN1* and *MME* were involved in the crassulacean pathway (CAM), and their expression levels were increased in P12h vs. H12h according to the FPKM values. Ribulose-1,5-bisphosphate carboxylase (Rubisco) as a key enzyme in the process of the Calvin cycle, its expression pattern was decreased (**Figure 4B**).

### Analyses of the DEGs related to glycolysis and the TCA cycle metabolic pathways in P12h and H12h

**Figure 5:**
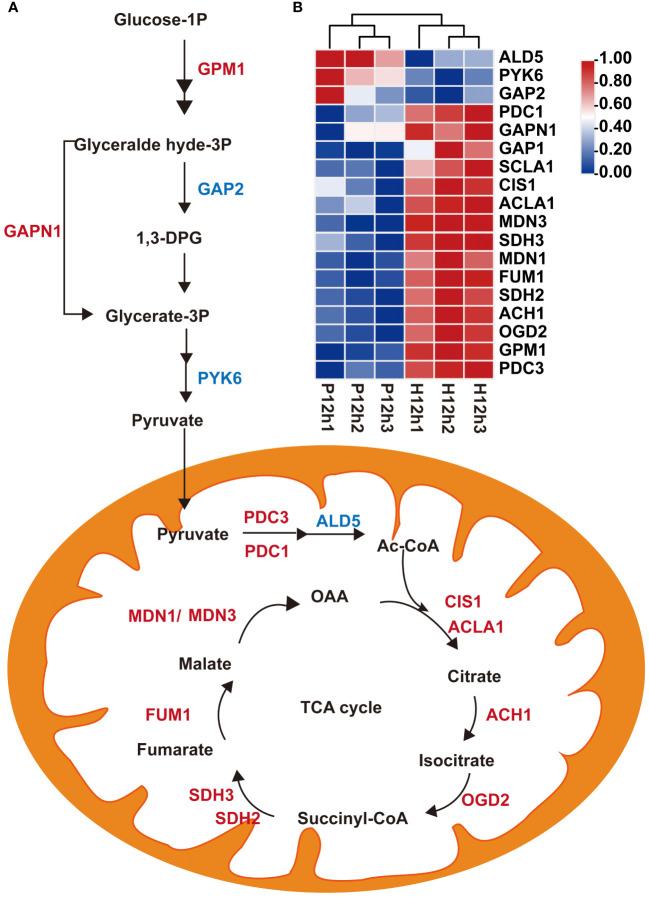
Differentially expressed genes (DEGs) between P12h and H12h were involved in glycolysis and the TCA cycle metabolic pathways. **(A)** Scheme of glycolysis and the TCA cycle in mitochondria of *C. reinhardtii*. 1,3-DPG, 1,3-diphosphoglyceric acid; Ac-CoA, acetyl-CoA; **(B)** The heatmap of relative expression of DEGs.

A total of 20 DEGs related to the glycolysis and the TCA cycle metabolic pathways were screened (**Supplementary Table S3**). A proposed glycolysis and the TCA cycle metabolic pathways were built based on these identified DEGs (**Figure 5A**). The expression levels of these DEGs were analyzed according to the FPKM values of transcriptome (**Figure 5B**). The expression levels of two key enzymes in glycolysis pathway phosphoglucomutase (GPM1) and glyceraldehyde 3-phosphate dehydrogenase (GAPN1) were up-regulated. However, the expression levels of three genes (*ALD5, PYK6*, and *GAP2*) were down-regulated from P12h to H12h (**Figure 5B**). Pyruvate, an intermediate of glycolysis, enters the tricarboxylic acid cycle (TCA). The expression levels of 11 key enzymes (PDC3, OGD2, ACH1, SDH2, FUM1, MDN1, SDH3, MDN3, ACLA1, CIS1 and PDC1) were significantly increased between P12h and H12h (**Figure 5**). The expression of most DEGs significantly increased from P12h to H12h in glycolysis and the TCA cycle metabolic pathways.

### Analyses of the DEGs related to pyruvate metabolic pathway in P12h and H12h

**Figure 6:**
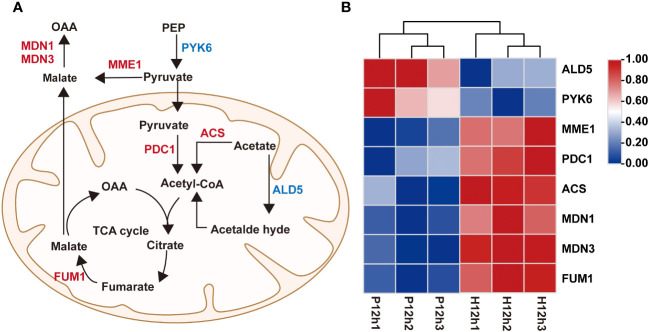
Differentially expressed genes (DEGs) between P12h and H12h were involved in pyruvate metabolic pathway. **(A)** Scheme of pyruvate metabolic network in mitochondria of *C. reinhardtii*. **(B)** The heatmap of relative expression of DEGs.

To understand the roles of DEGs in regulating pyruvate metabolic pathway, correlation analysis between pyruvate metabolic network and the expression profiles of the DEGs were performed (**Figure 6**). In this study, a total of 8 DEGs were identified to be implicated in pyruvate metabolic pathway, including *ALD5, PYK6, MME1, PDC1, ACS, MDN1, MDN3* and *FUM1* (**Figure 6A** and **Supplementary Table S4**). According to the FPKM values of transcriptome, the expression of *ALD5* and *PYK6* gradually decreased from P12h to H12h. By contrast, the expression levels of *MME1, PDC1, ACS, MDN1, MDN3* and *FUM1* were dramatically up-regulated (**Figure 6B**).

### Analyses of the DEGs related to oxidative phosphorylation in P12h and H12h

**Figure 7:**
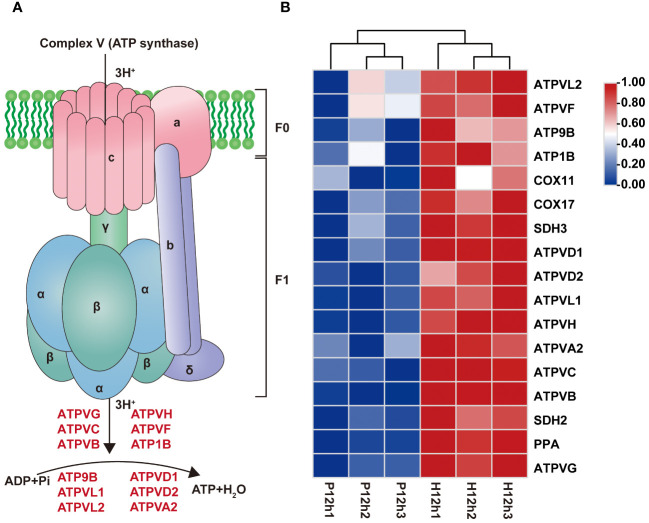
Differentially expressed genes (DEGs) between P12h and H12h were involved in oxidative phosphorylation. **(A)** Schematic diagram showing ATP synthase. ATP synthase structural model was constructed (Ge et al., 2021). **(B)** The heatmap of relative expression of DEGs related to oxidative phosphorylation.

According to transcriptome results, the 17 DEGs involved in oxidative phosphorylation were significantly up-regulated from P12h to H12h (**Figure 7**; **Supplementary Table S5**). Based on previous research we constructed a diagram of complex V (ATP synthase) which was a key enzyme in oxidative phosphorylation (**Figure 7A**). Moreover, the 12 genes were summarized together with ATPase for catalyze ATP synthesis (**Figure 7B**). Transcriptome data revealed that expression levels of these genes were significantly increased in P12h vs. H12h by the FPKM values of transcriptome (**Figure 7B**).

### The relative expressions of marker genes

**Figure 8:**
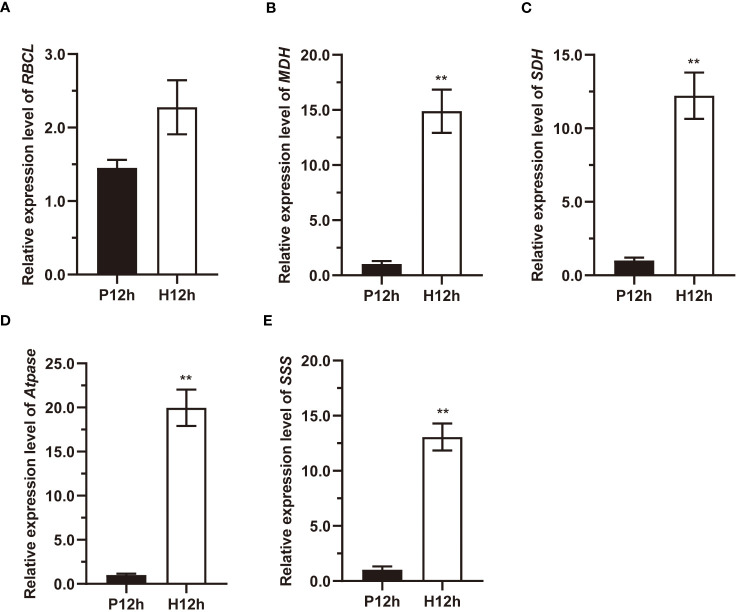
qPCR expression profile of marker genes *RBCL, MDH, SDH, Atpase, and SSS* between the P12h and the H12h groups. **(A)** Expression changes of *RBCL*; **(B)** Expression changes of *MDH*; **(C)** Expression changes of *SDH*; **(D)** Expression changes of *ATPase*; **(E)** Expression changes of *SSS*. Data are representative of three independent experiments. Significance was determined by t-test analysis: ** indicated P < 0.01.

To further explore the relationship between DEGs and the major metabolic pathways mentioned above, the relative expression levels of *RBCL, MDH, SDH, Atpase*, and *SSS* were detected by qRT-PCR (**Figure 8**). Detailed analysis indicated that ribulose-1,5-bisphosphate carboxylase (RBCL) transcripts were increased from P12h to H12h (**Figure 8A**). The relative expression patterns of *MDH, SDH, ATPase* and *SSS* were increased significantly between P12h and H12h. As shown in **Figure 8B**, relative expression levels of NAD-malate dehydrogenase (MDH) in the H12h group were significantly higher than in the P12h group. **Figure 8C** demonstrated that the expression of succinate dehydrogenase (SDH) in the P12h group was 1.0120 and dramatically increased to 12.2199 in the H12h group. In P12h vs. H12h, the expression of ATP synthase (ATPase) increased significantly (P< 0.05) (**Figure 8D**). The expression of starch synthase (SSS) increased from only 1.0319 in the P12h group to 13.0615 in the H12h group (**Figure 8E**). These results were consistent with RNA-seq.

### The changes of key enzyme activities in P12h and H12h

**Figure 9:**
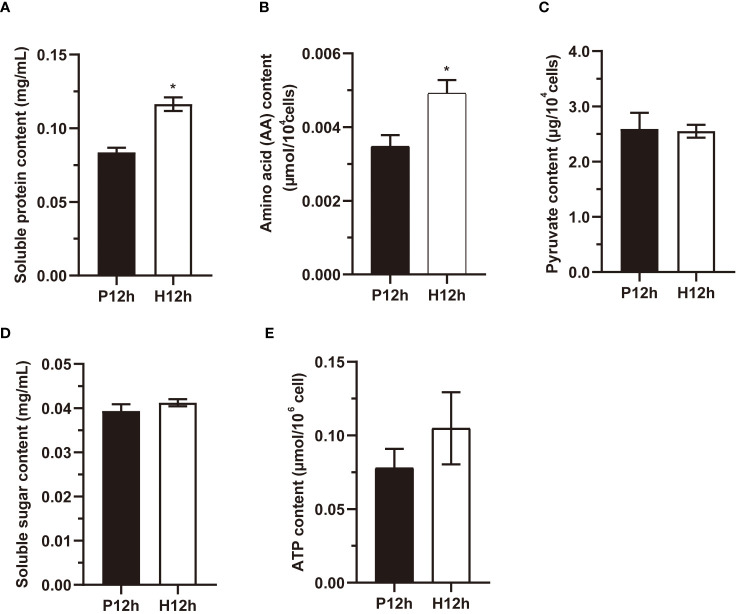
The key enzyme activities Rubisco, NAD-MDH, SSS, PK, and SDH in the P12h and the H12h groups. **(A)** Rubisco activity in P12h and H12h. **(B)** NAD-MDH activity; **(C)** SSS activity; **(D)** PK activity; **(E)** SDH activity. Data are representative of 3 independent experiments. Significance was determined by t-test analysis: * indicated P < 0.05.

To comprehend the roles of key DEGs in the regulation of main metabolic pathways, the changes of key enzyme activities were examined. The activity of Rubisco showed a significant decline (p< 0.01) from the P12h group to the H12h group (**Figure 9A**). In contrast, the NAD-MDH activity of the H12h group was significantly higher than the P12h group (**Figure 9B**). The changes of SSS activity and pyruvate kinase activity (PK) were no significant among two groups, presented in **Figures 9C**, **9D**. Activity of the SDH was markedly increased (p< 0.01) from the P12h group to the H12h group (**Figure 9E**).

## Discussion

### Photoautotrophy and heterotrophy affect the growth of *C. reinhardtii*

The photoautotrophic growth is an attractive cultivation because it can be used for natural resources, for example, sunlight and carbon dioxide. Even though photosynthetic autotrophy has these advantages, the maximum biomass concentrations can be negatively correlated with light penetration (Richmond, 2013). The heterotrophy of microalga is cultivated in the dark where the cell growth and reproduction are supported by organic carbons (Chen and Jiang, 2017). It has been reported that heterotrophic growth of the diatom *Nitschia laevis*, the dinoflagellate *Crypthecodinium cohnii*, and the rhodophyte *Galdieria sulphuraria* accumulated much higher cell densities and biomass productivities than autotrophic growth cell (De Swaaf et al., 2003; Wen and Chen, 2003; Schmidt et al., 2005; Graverholt and Eriksen, 2007). The previous results indicated that cultivation conditions had significant effects on *C. reinhardtii* biomass productivity and bioproduct accumulation (Pessoa et al., 2023). It demonstrated that the biomass, carbohydrates, and chlorophyll b production were higher in photoautotrophic growth. In contrast, lipids and chlorophyll concentrations were higher in heterotrophic growth (Boyle and Morgan, 2009b). Our results represented that Fv/Fm and total chlorophyll content of photoautotrophy were significantly higher than heterotrophy (**Figure 1**). Our results have the same findings as previous studies. The previous studies reported that the chlorophyll content of *Nannochloropsis* and *Chlorella* were reduced in heterotrophic conditions, compared with autotrophic conditions (Cheirsilp and Torpee, 2012).

### Transcriptomics reveals the expression of DEGs from autotrophs to heterotrophs

In previous studies, the transcriptome was widely used in heterotrophic and autotrophic microalga, including physiology and metabolism, development and life cycles, environmental and ecological studies (Anderson, 2022b). In this study, the transcriptomics analysis was estimated for samples from photoautotrophy (P12h) and heterotrophy (H12h) (**Figure 2**). Heatmap showed the FPKM expression patterns that were normalized, mean-centered, and scaled by Z-score method. In the bubble chart of KEGG enrichment, amino acid metabolic pathways and some glycol-metabolism pathways were enriched in H12h vs. P12h (**Figure 3**). Above results suggested that these metabolic pathways presumably were involved in the growth of *C. reinhardtii* under heterotrophy.

### Functional analysis of DEGs in main metabolic pathways

Further functional evaluation of DEGs was performed by analyzing the important metabolic pathways, including photosynthesis carbon fixation, glycolysis, the TCA cycle, pyruvate metabolism, and oxidative phosphorylation. The Calvin cycle is an important process of carbon fixation in photosynthesis which chloroplasts convert air-borne CO_2_ into carbohydrates (Yang et al., 2017). In this study, there were 9 DEGs involved in the photosynthesis and carbon fixation metabolic pathways, which were increased in heterotrophy, as shown in **Figure 5**. Heterotrophic cultures accelerated the photosynthetic carbon fixation process, providing cell with more 3-phosphoglycerate for cellular metabolic processes that may consume excess ATP and NADPH than autotrophic conditions.

Pyruvate is the main product of cytosolic glycolysis and the primary respiratory substrate for energy production to support cell growth in most organisms (Le et al., 2022). It is first oxidized by the mitochondrial pyruvate dehydrogenase complex (PDC) then produces acetyl-CoA which enters the tricarboxylic acid cycle (TCA), finally, generates reductant equivalent for ATP production (Day and Hanson, 1977; Le et al., 2021). The transcriptome data analysis revealed alterations of gene expression of the enzyme coding genes from major metabolic pathways, including Glycolysis, Pyruvate metabolism, and Tricarboxylic acid cycle (TCA) (**Figures 5**, **6**). Several alterations in the expression of enzymes point that TCA may exert an important role in accelerating or increasing the production of citrate and malate in heterotrophy.

Oxidative phosphorylation of cell is the main form of energy generation to maintain vital life activities. There are four main membrane-bound complex I (Hatefi et al., 1979), complex II (Lemire and Oyedotun, 2002), complex III (Tsukihara et al., 1996; Zhang et al., 1998), and complex IV (Poyton et al., 1995) participate in mitochondrial oxidative phosphorylation. In bacteria, ATP synthase consists of eight subunits, α_3_β_3_γδϵab_2_c_n_, and F_1_ corresponds to α_3_β_3_γδϵ and F_0_ to ab_2_c_n_ (Weber and Senior, 2003). Transcriptomic results demonstrated that 12 genes were involved in ATP synthesis and the expression levels were dramatically raised from P12h to H12h in **Figure 7**. These differences between photoautotrophic and heterotrophic conditions indicated that the cell may transport excess electrons through cyclic electron transport chain under heterotrophic conditions. The genes involved in ATPase were activated in heterotrophic conditions, indicating that ATP was required for cell phosphorylation.

### Molecular mechanism through which DEGs regulate between P12h and H12h

In this study, the activity and expression levels of key enzymes were analyzed in the above major metabolic pathways. RBCL is the major soluble protein in plants, which catalyzes the initial step in Calvin’s reductive pentose phosphate cycle (Miziorko and Lorimer, 1983). MDH plays a crucial role in several metabolic pathways, including the TCA cycle, glyoxylate bypass, amino acid synthesis, and so on (Musrati et al., 1998). SDH catalyzes the oxidation of succinate to fumarate and thereby link the TCA cycle (Jardim-Messeder et al., 2015). The transcripts of *MDH, SDH, Atpase*, and *SSS* were significantly increased during the H12h treatment, as shown in **Figure 8**. These differences between autotrophic and heterotrophic conditions indicated that glycolysis and the TCA cycle, oxidative phosphorylation, and pyruvate metabolisms were activated in heterotrophic conditions.

According to previous studies, the autotrophic, mixotrophic, and heterotrophic phenotypes in the microalga *Chlorella vulgaris* was compared by using proteomics and transcriptomics, indicating that myo-inositol may exert a signaling and regulatory role over the cell growth performance of *C. vulgaris* in mixotrophy (Vidotti et al., 2020). However, there were limited publications that discussed the potential growth differences of *C. reinhardtii* under autotrophic and heterotrophic conditions. In present study, we examined pivotal indicators related to the growth of *C. reinhardtii* under autotrophic and heterotrophic cultures, in which the P12h group had significantly higher cell numbers than the H12h group. Moreover, the transcriptome and molecular analysis indicated that the expression of genes in major metabolisms (glycolysis, tricarboxylic acid cycle, pyruvate metabolism, and oxidative phosphorylation) were activated in heterotrophy. To gain more insight into the regulation of metabolic pathways, we will identify novel candidate genes in heterotrophy and photoautotrophy to explain regulatory mechanisms of cell growth.

## Conclusion

Heterotrophic cultures of *C. reinhardtii* had a significantly higher cell count than autotrophic conditions. Our findings showed enhanced photosynthetic carbon fixation, the TCA cycle, pyruvate and oxidative phosphorylation metabolisms, which ensured an efficient supply of energy and carbon skeleton for rapid growth of *C. reinhardtii* under heterotrophic conditions. Therefore, the results of this study provided a theoretical basis and an experimental basis for the growth of *C. reinhardtii* in photoautotrophy and heterotrophy.

## Data availability statement

The datasets presented in this study can be found in online repositories. The names of the repository/repositories and accession number(s) can be found in the article/**Supplementary Material**.

## Ethics statement

*Chlamydomonas reinhardtii* strain CC-125 was obtained from the Chlamydomonas Resource Center (CRC, University of Minnesota). The study complies with all relevant institutional, national, and international guidelines and legislation in the field.

## Author contributions

JC: Writing – original draft, Writing – review & editing. YC: Investigation, Resources, Software, Validation, Visualization, Writing – review & editing. WH: Data curation, Formal analysis, Writing – review & editing. HL: Investigation, Methodology, Writing – original draft. TH: Data curation, Methodology, Writing – review & editing. TL: Supervision, Writing – original draft. HD: Resources, Supervision, Writing – review & editing.
