# Supplementary material for: Transcriptome analysis reveals the molecular mechanism of differences in growth between photoautotrophy and heterotrophy in Chlamydomonas reinhardtii
Source: Front Plant Sci. 2024 Jun 19;15:1407915. doi: 10.3389/fpls.2024.1407915 (PMC11219824; doi:10.3389/fpls.2024.1407915)
Supplement: Supplementary file 1 [file DataSheet_1.docx]

**Transcriptome analysis reveals the molecular mechanism of differences in growth between photoautotrophy and heterotrophy in *Chlamydomonas reinhardtii***


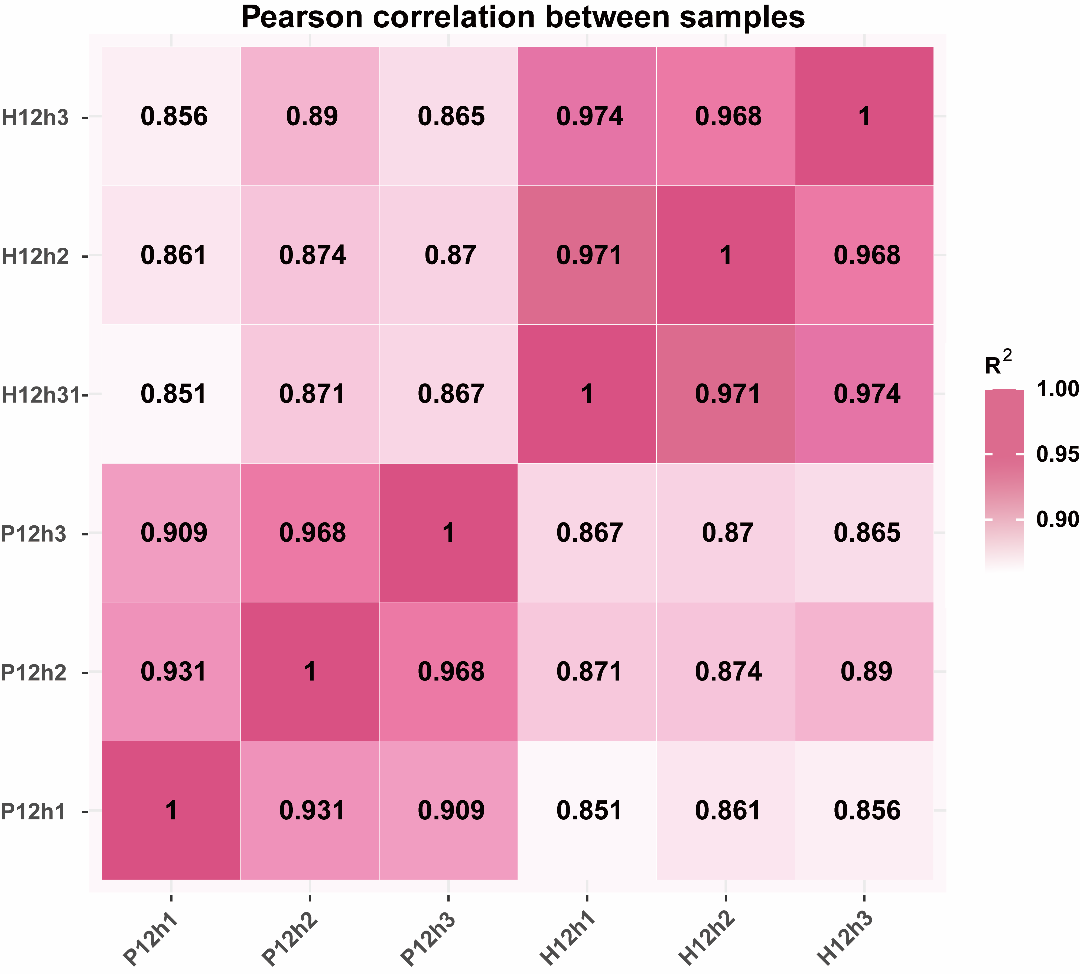
**Supplementary Figures**

Supplementary Fig. S1. Correlation coefficients for parallel samples. The horizontal and vertical coordinates in the graph are the squares of the correlation coefficients for each sample.


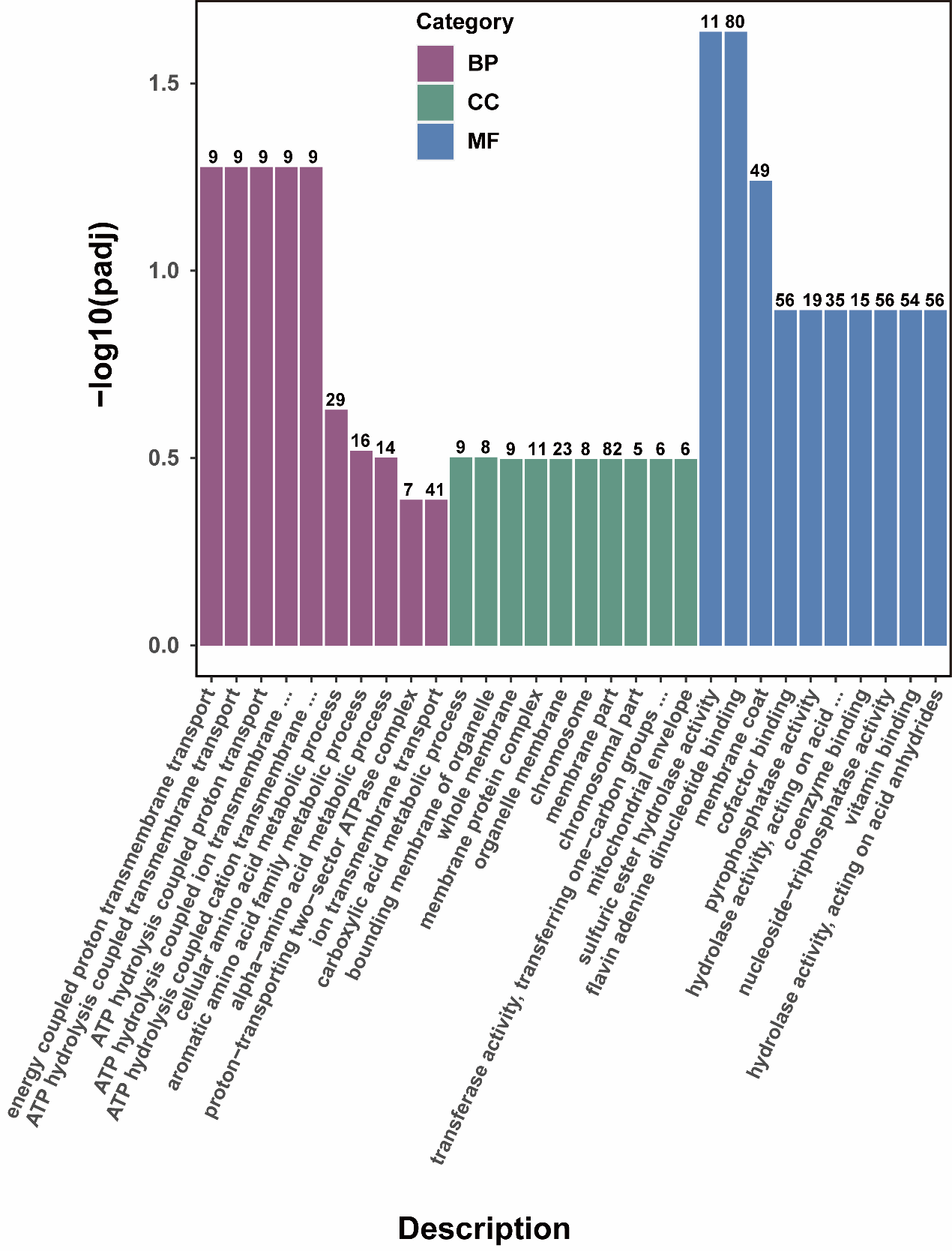


Supplementary Fig. S2. GO functional classification analysis for the DEGs from H12h vs. P12h. The horizontal coordinate of the graph is GO Term, the vertical coordinate is the significance level of GO Term enrichment, represented by -log10 (padj), and different colors indicate different functional classifications, respectively.


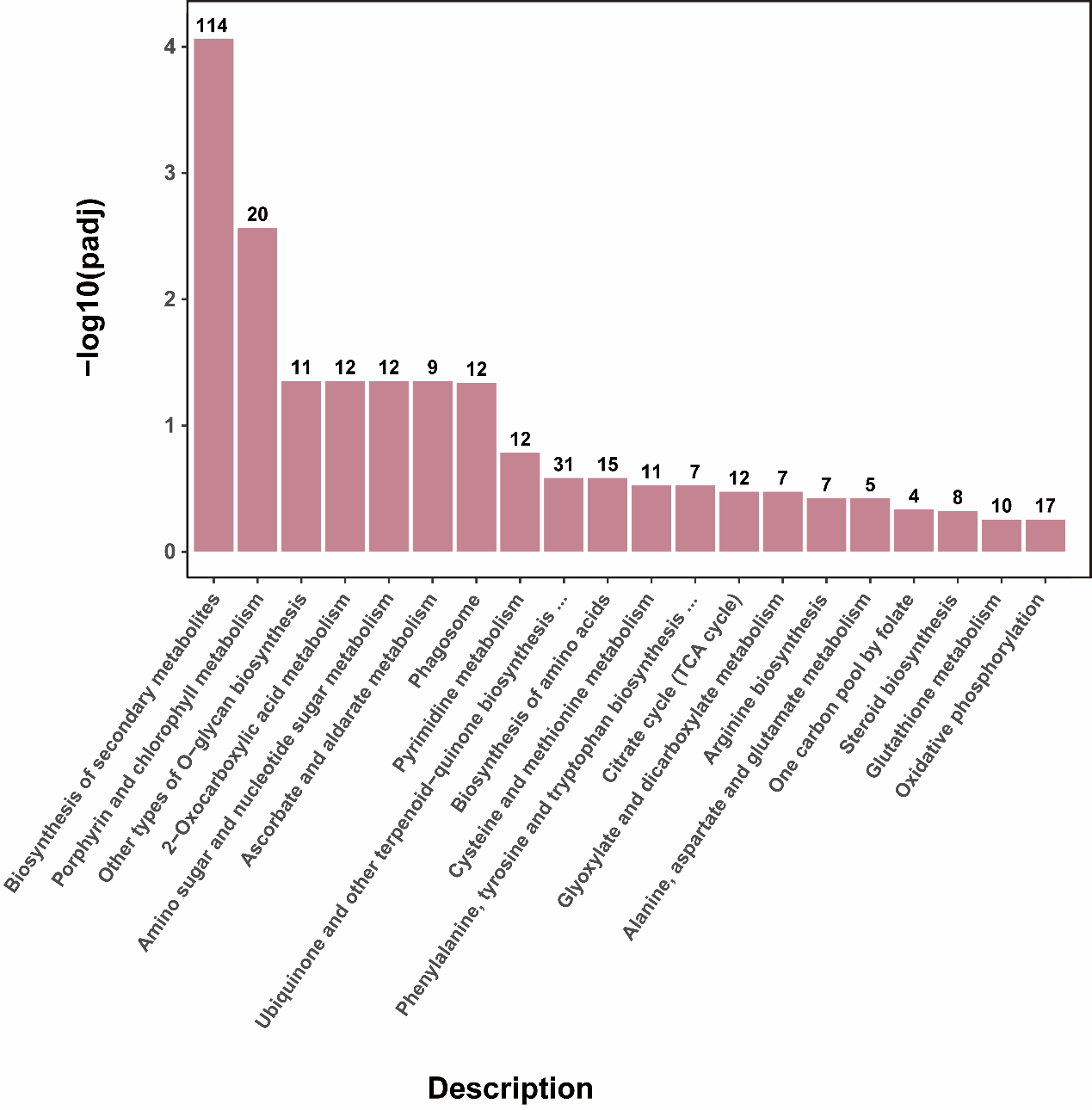


Supplementary Fig. S3. KEGG pathway enrichment analysis for the DEGs from H12h vs. P12h. The x-axis represents the top 20 KEGG pathways and the y-axis represents the number of identified metabolites involved in this pathway.
