# Supplementary material for: Transcriptome analysis reveals the molecular mechanism of differences in growth between photoautotrophy and heterotrophy in Chlamydomonas reinhardtii
Source: Front Plant Sci. 2024 Jun 19;15:1407915. doi: 10.3389/fpls.2024.1407915 (PMC11219824; doi:10.3389/fpls.2024.1407915)
Supplement: Supplementary file 2 [file DataSheet_2.docx]

Supplementary Table S1. Summary statistics of the transcriptome of *C. reinhardtii*.

| **Sample name** | **Raw reads** | **Clean reads** | **Q30 (%)** | **Total mapped reads** | **Mapping rate (%)** | **Total genes (FPKM>1)** |
| --- | --- | --- | --- | --- | --- | --- |
| P12h1 | 47814442 | 47637228 | 92.84 | 45174805 | 94.83% | 12837 |
| P12h2 | 47100546 | 46024520 | 93.8 | 44733105 | 97.19% | 12506 |
| P12h3 | 40268920 | 38180774 | 94.29 | 36749621 | 96.25% | 12465 |
| H12h1 | 40055138 | 37567000 | 94.19 | 35933876 | 95.65% | 12350 |
| H12h2 | 39836978 | 37876526 | 94.14 | 36437601 | 96.20% | 12214 |
| H12h3 | 45877408 | 44833684 | 93.81 | 43270504 | 96.51% | 12192 |

Supplementary Table S2. Differential gene names marked with red and blue correspond to the gene symbol in the heatmap of photosynthesis and carbon fixation metabolic pathway in Fig. 4.

| **Gene ID** | **Gene name** |
| --- | --- |
| CHLRE_06g257950v5 | AST |
| CHLRE_02g145800v5 | MDN2 |
| CHLRE_03g194850v5 | MDN1 |
| CHLRE_06g268750v5 | MME |
| CHLRE_10g451950v5 | AAT |
| CHLRE_12g485150v5 | GAP1 |
| CHLRE_02g120150v5 | Rubisco |
| CHLRE_03g183850v5 | FDX |
| CHLRE_01g016750v5 | PSBS |
|  |  |

Supplementary Table S3. Differential gene names marked with red and blue correspond to the gene symbol in the heatmap of the glycolysis and the TCA cycle metabolic pathway in Fig. 5.

| **Gene ID** | **Gene name** |
| --- | --- |
| CHLRE_06g278210v5 | GPM1 |
| CHLRE_03g165700v5 | PDC3 |
| CHLRE_12g500150v5 | ALD5 |
| CHLRE_12g485150v5 | GAP1 |
| CHLRE_07g337650v5 | PDC1 |
| CHLRE_12g556600v5 | GAPN1 |
| CHLRE_10g426292v5 | PYK6 |
| CHLRE_07g354200v5 | GAP2 |
| CHLRE_01g042750v5 | ACH1 |
| CHLRE_02g145800v5 | MDN3 |
| CHLRE_03g194850v5 | MDN1 |
| CHLRE_06g254400v5 | FUM1 |
| CHLRE_07g343700v5 | OGD2 |
| CHLRE_01g020350v5 | SDH3 |
| CHLRE_06g264200v5 | SDH2 |
| CHLRE_03g193850v5 | SCLA1 |
| CHLRE_07g337650v5 | PDC1 |
| CHLRE_12g514750v5 | CIS1 |
| CHLRE_05g241850v5 | ACLA1 |

Supplementary Table S4. Differential gene names marked with red and blue correspond to the gene symbol in the heatmap of the pyruvate metabolic pathway in Fig. 6.

| **Gene ID** | **Gene name** |
| --- | --- |
| CHLRE_02g145800v5 | MDN3 |
| CHLRE_03g194850v5 | MDN1 |
| CHLRE_06g254400v5 | FUM1 |
| CHLRE_06g268750v5 | MME1 |
| CHLRE_01g055408v5 | ACS |
| CHLRE_12g500150v5 | ALD5 |
| CHLRE_07g337650v5 | PDC1 |
| CHLRE_10g426292v5 | PYK6 |

Supplementary Table S5. Differential gene names marked with red and blue correspond to the gene symbol in the heatmap of the glycolysis and the TCA cycle metabolic pathway in Fig. 7.

| **Gene ID** | **Gene name** |
| --- | --- |
| CHLRE_09g387875v5 | PPA |
| CHLRE_11g468550v5 | ATPVG |
| CHLRE_01g020350v5 | SDH3 |
| CHLRE_06g250250v5 | ATPVC |
| CHLRE_09g402500v5 | ATPVA2 |
| CHLRE_06g264200v5 | SDH2 |
| CHLRE_06g257450v5 | ATPVL1 |
| CHLRE_02g076350v5 | ATPVB |
| CHLRE_01g027800v5 | ATPVH |
| CHLRE_03g176250v5 | ATPVD1 |
| CHLRE_05g232850v5 | COX17 |
| CHLRE_17g731950v5 | ATP9B |
| CHLRE_09g405050v5 | ATPVD2 |
| CHLRE_12g529100v5 | ATPVL2 |
| CHLRE_10g419050v5 | ATP1B |
| CHLRE_01g055550v5 | COX11 |
| CHLRE_10g446550v5 | ATPVF |

Supplementary Table S6. Oligonucleotides were used in this work for quantitative RT-PCR experiments.

| **Gene symbol (NCBI)** | **Gene ID (NCBI)** | **Gene ID**  **(JGI _Chlamydomonas reinhardtii v5.6)** | **Name** | **Oligo** |
| --- | --- | --- | --- | --- |
| CHLRE_03g159200v5 | 5723012 | Cre03.g159200.t1.1 | UBC8-F | GTACAGCGGCGGCTAGAGGCAC |
|  |  |  | UBC8-R | AGCGTCAGCGGCGGTTGCAGGTATCT |
| HLRE_01g020350v5 | 5715192 | Cre01.g020350.t1.2 | SDH-F | ATGCAGCGGTCAAGTCG |
|  |  |  | SDH-R | TCACAGCACCGCGG |
| CHLRE_02g120150v5 | 5727948 | Cre02.g120150.t1.2 | RBCL-F | ATGGCCGCCGTCATTGC |
|  |  |  | RBCL-R | TTACACGGAGCGCTTGTTGG |
| CHLRE_03g194850v5 | 5718730 | Cre03.g194850.t1.2 | MDH-F | ATGCTGCTCGCGAACC |
|  |  |  | MDH-R | GGCTCTTGACAAAGTCGAGG |
| CHLRE_09g402500v5 | 5720728 | Cre09.g402500.t1.2 | Atpase-F | GATTTTGGCTTCCAGAACATTGAC |
|  |  |  | Atpase-R | CAGCTGCTTCAGGGTCG |
